# Huge abdominal cerebrospinal fluid pseudocyst following ventriculoperitoneal shunt: a case report

**DOI:** 10.1186/s13256-019-2308-0

**Published:** 2019-12-10

**Authors:** Yasuhiro Koide, Takaaki Osako, Masahiro Kameda, Hiromi Ihoriya, Hirotsugu Yamamoto, Noritomo Fujisaki, Toshiyuki Aokage, Tetsuya Yumoto, Isao Date, Hiromichi Naito, Atsunori Nakao

**Affiliations:** 1 Department of Emergency, Critical Care and Disaster Medicine, Okayama University Graduate School of Medicine, Dentistry and Pharmaceutical Sciences, Okayama, Japan; 2 Center for Graduate Medical Education, Okayama University Hospital, Okayama, Japan; 3 Department of Neurological Surgery, Okayama University Graduate School of Medicine, Dentistry and Pharmaceutical Sciences, Okayama, Japan

**Keywords:** Abdominal pseudocyst, Cerebrospinal fluid, Complication, Ventriculoperitoneal shunt

## Abstract

**Introduction:**

Abdominal pseudocysts comprising cerebrospinal fluid are an uncommon but significant complication in patients with ventriculoperitoneal shunt. We present a successfully treated 12-year-old boy with a history of ventriculoperitoneal shunting and a huge abdominal cerebrospinal fluid pseudocyst.

**Case presentation:**

A12-year-old Japanese boy presented with a deteriorated consciousness and a palpable and elastic large lower abdominal mass. Computed tomography of his abdomen demonstrated a collection of homogenous low-density fluid near the catheter tip of the ventriculoperitoneal shunt. Cerebral computed tomography revealed an increased ventricular size. Based on the clinical diagnosis of abdominal pseudocyst, the peritoneal shunt catheter was secured and divided into two parts by cutting it on the chest; then, the proximal side of the peritoneal shunt catheter was externalized for extraventricular drainage. The cyst was percutaneously aspirated with ultrasound guidance, and the distal side of the peritoneal shunt catheter was removed. The distal side of the peritoneal shunt catheter was reinserted in another position into his abdomen after 3-week extraventricular drainage management.

**Conclusion:**

Emergency physicians should know about this potential complication as an important differential diagnosis resulting from acute abdominal complaints in patients with ventriculoperitoneal shunts.

## Introduction

Placement of a ventriculoperitoneal (VP) shunt is a common surgical procedure used to manage hydrocephalus for cerebrospinal fluid (CSF) diversion. Abdominal pseudocysts are relatively rare abdominal complications resulting from VP shunt insertion [1–3]. Here, we describe a 12-year-old patient with a VP shunt placed for congenital hydrocephalus presenting vomiting, cephalalgia, apathy, and an indurated abdominal mass with no fever. Sharing our experience and typical radiographic images may help improve emergency physicians’ awareness of this important complication and decisions on the best therapeutic strategy.

**Fig. 1:**
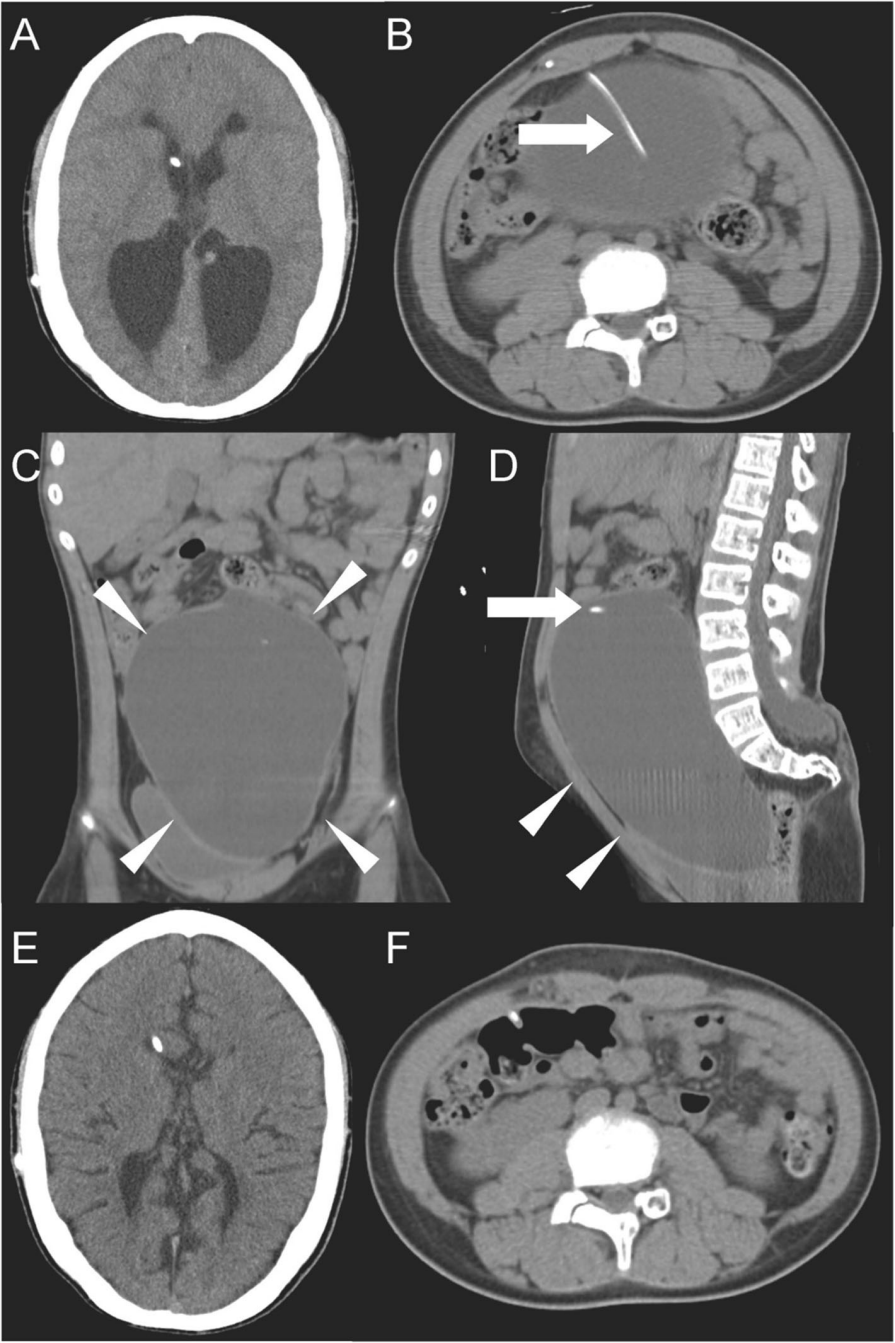
Cranial computed tomography demonstrated bilateral ventricular dilatation, periventricular edema, and effacement of sulci (**a**). Abdominal computed tomography demonstrated a cyst comprising a collection of homogeneous low-density fluid in the abdominal cavity with displacement of the bowel loops to the posterior part. The ventriculoperitoneal shunt catheter tip was located adjacent to the collection of fluid (**b** axial, **c** coronal, **d** sagittal). Computed tomography obtained before revision of the distal side of the peritoneal shunt catheter indicated improvement of the hydrocephalus (**e**). Computed tomography obtained after revision of the distal side of the peritoneal shunt catheter indicated no recurrence of the pseudocyst (**f**). The cyst and the shunt catheter are indicated by *white arrowheads* and *arrows*, respectively

## Case presentation

A 12-year-old Japanese boy presented with complaints of disturbance of consciousness and vomiting associated with increasing abdominal distension starting 3 days prior to presentation. He complained of headache and appetite loss for 7 days. His previous medical history included repair of myelomeningocele on the day of his birth; subsequently, a VP shunt was placed for congenital hydrocephalus associated with spina bifida when he was 10-days old. Thereafter, he underwent several subsequent abdominal surgeries for reinsertion of the shunt catheter and was followed up every 6 months without medication. A physical examination revealed a deteriorated consciousness (Glasgow Coma Scale score of E1V2M4) and a large palpable elastic mass in his lower abdomen. His vital signs were stable (blood pressure 124/76 mmHg, heart rate 88 beats/minute, body temperature 36.8 °C). His pupil sizes were 3 mm in each eye with rapid light reflex. Laboratory tests results revealed an increased white blood cell count of 9910/μL (3300–8600 /μL) and a C-reactive protein level of 0.54 mg/dL (< 0.15 mg/dL). Liver and kidney functions were normal with normal serum electrolytes. Serum ammonia level was 34 μg/dL (30–80 μg/dL). Urine analysis results were unremarkable. An abdominal ultrasound confirmed a large collection of homogeneous encysted fluid encapsulating the tip of the shunt catheter. Abdominal radiography showed the VP shunt catheter and the presence of a soft tissue mass in his upper abdomen. Cranial computed tomography (CT) showed bilateral ventricular dilation and effacement of sulci. An abdominal CT scan demonstrated a 11 cm × 8 cm × 7 cm collection of homogenous low-density fluid adjacent to the catheter tip of the VP shunt (Fig. 1). As abdominal pseudocyst associated with shunt malfunction was highly suspected as a diagnosis, the proximal side of the peritoneal shunt catheter (Strata® 0.5, NSC™ valve, Medtronic, Inc., Minneapolis, MN, USA) was distally externalized for extraventricular drainage management by neurosurgery. Subsequently, the cystic mass was punctured through the abdominal wall and 900 mL of clear fluid was drained. Culture of the drained intracystic fluid was negative for microorganisms, with protein 36 IU/L and glucose 71 mg/dL. The leukocyte and erythrocyte counts were 0 and 2/μL, respectively. The distal side of the peritoneal shunt catheter was removed from his abdominal cavity. Prophylactic cefazolin sodium 3 grams/day was administered for 3 days postoperatively. A follow-up brain CT taken 3 days postoperatively revealed no increase in ventricular size. The shunt catheter was reinserted to another position in his abdomen 3 weeks later. He fully recovered with no further complications.

## Discussion

Abdominal CSF pseudocyst is a relatively rare VP shunt complication, but should be considered in all patients with abdominal symptoms or intracranial hypertension signs. Differential diagnoses of an abdominal cystic mass include: benign cystic teratoma; full-term pregnancy; mesenteric abscess; cystic lymphangioma; seroma; lymphocele; pancreatic pseudocyst; and mesenteric, duplication, and ovarian cysts [4, 5]. Although the etiology of a CSF pseudocyst is not well known, the incidence is reported to range from less than 0.33 to 6.8% [6]. The time between last VP shunt surgery and abdominal CSF collection ranges from 3 weeks to 21 years [7]. In addition to abdominal CSF pseudocyst, relatively uncommon complications include perforation of the small bowel with secondary cerebrospinal-enteric fistula, subphrenic abscess, movement of the shunt tip to distant locations like the subphrenic or intrathoracic areas, untreatable CSF ascites, and shunt protrusion from the anus [8].

Pathogenesis of CSF pseudocyst is at least in part related to the prior abdominal surgery and liver dysfunction [9], as well as infections and allergic reactions to silicone or ethylene oxide [10]. Potential organisms that are implicated in CSF pseudocysts include *Staphylococcus epidermidis, Staphylococcus aureus,* and *Propionibacterium acnes* [11]. In the present case, no microorganism growth was observed in the cultures of the cyst fluid and the distal shunt tip. We assume that the pseudocyst in our case developed primarily due to peritoneal adhesions associated with the repeat revisions.

In addition to shunt malfunction signs like lethargy and headache, typical clinical presentation includes abdominal pain and/or palpable abdominal mass, distention, nausea, vomiting, and decreased appetite and fever, resembling acute abdomen [12, 13]. Diagnosing abdominal pseudocyst depends on access to the patient’s detailed medical history and compelling clinical suspicion. In patients with signs of intracranial hypertension, cerebral CT can be used to assess the ventricular dilatation, shunt position, and effacement of sulci. Ultrasonography has proved to be the preferred method to diagnose abdominal complications resulting from VP shunting, especially CSF pseudocyst. CT can also reliably confirm a pseudocyst. A CT scan is often more useful in distinguishing between etiologies presenting with severe abdominal pain such as appendicitis, diverticulitis, abdominal abscess, or bowel obstruction. Typical CT findings used to diagnose abdominal pseudocyst include collection of intraperitoneal fluid with clear margins, generally without internal septa, and recognition of the distal tip of the shunt catheter inside or adjacent to the pseudocyst.

The peritoneal cavity remains the most common site for successful CSF diversion because of the low incidence of serious complications, since the peritoneal cavity is the best site for CSF absorption [14]. If the patient’s peritoneal condition allows it, experts recommend a laparoscopic procedure to avoid development of a peritoneal adhesion, which increases CSF pseudocyst recurrence [15]. If an infection is present, the shunt is externalized; a CSF culture should be administered, and systemic antibacterial therapy should be adjusted to the results of the culture. In an emergency setting, image-guided aspiration of an abdominal pseudocyst may be used to alleviate acute symptoms while awaiting elective revision of the shunt [16]. Thus, pseudocyst treatment varies and no standards have been established; treatment strategies should be adjusted for the patient’s overall clinical status. The risks of complications associated with VP shunt vary with the patient’s size and condition, operating surgeon’s experience, and use of prophylactic antibiotics. Recognizing these facts and consequent treatment changes may result in improvement of patient outcomes in those undergoing VP shunt placement [3].

## Conclusion

Every physician caring for patients with VP shunts should be aware of the complication of abdominal pseudocysts following VP shunting.

## Data Availability

None.

## Abbreviations

CSF: Cerebrospinal fluid
CT: Computed tomography
VP: Ventriculoperitoneal
